# Zeutralblott feur Chirurgie

**Published:** 1912-11

**Authors:** 


### Zeutralblott feur Chirurgie

No. 46, Nov. 18, 1911.—Franz Fink reports a case of Gastric Ulcer symptomatically cured by gastero-enterostomy for 2 years. Then recurrence. At a second operatioin the gastro-enterostomy opening was found to have closed through the action of adhesions. After resection of the ulcer again recovery, one year to date.

Reports from the convention of German Natural Scientists and Physicians. September, 1911. Stoffel treats spastic paralysis by selective neurotomy in the distribution of the spastic muscles thereby weakening these sufficiently to restore the balance with the sound muscles.

No. 50.—From the same reports. Wilms reports two successful cases wherein he used a rubber drain to replace the common bile duct, leaving it in situ covered by omentum or the colon. He recommends this where other measures seem impossible.

No. 50, December 16.—Meeting of German-Northwestern Association of Surgeons, October, 1911. Brewitt of Lubeck reports seventeen cases of urethral stricture treated more successfully by electrolysis than by dilatation, three or four treatments of a few minutes each with a two milliampere current being the average course.

No. 2, January 13, 1912.—Pressure paralysis following Esmarck Constriction. Wilhelm Wolf discusses recently reported cases. He deprecates wholesale condemnation of the measure in view of the fact that practically all such cases ultimately recover. Besides careless application of the bandage he suggests longstanding syphilis as responsible for this accident in most cases.

No. 4, January 27, 1912.—Gastric motor insufficiency due to Perigastritis of Gonorrhoeal origin. I. I. Grepow, argues "post hoc" that adhesions about the pylorus with symptoms, as a sequel to pelvic disturbances are of gonorrhoeal origin. He cites two cases operated upon by himself which showed the effects of perigastritis, but he brought forward only the history of the case to prove gonorrhoeal origin—a plausible but by no means conclusive argument.

No. 5, February 3.—C. Hubscher recommends hourly exercises in situ to prevent muscle atrophy following injuries to extremeties requiring plaster splints.

### Experimental Graves Disease

No. 5, February 3.—Bircher claims to have elicited typic symptoms in dogs by intraperitoneal implantation of pieces of thymus gland, freshly removed from living patients with enlarged thymus, or from those recently dead during narcosis in whom enlarged thymus was found.

No. 10, March 9.—Max Boruch produced experimental Graves Disease by injection of fresh ground human thyroid in of using expressed thyroid juices for Graves Diseases, criticize type.

No. 19, May 11.—Klose & Lampi, in defense of their method of using expressed thyroid juices for Graves Diseases, criticize above methods as regards propriety of results and accuracy of dosage.

No. 27, July 6.—Boruch, in rebuttal vs. Klose & Lampi.

No. 9, March 2.—Laminectonny under Local Anaesthesia. L. Heidenhain reports having done numerous major operations under local anaesthesia after Braun. Preliminary morphia than 1-2 per cent. novocain with suprarenin. He reports in detail a tre-panation for brain abscess and a multiple laminetomy by the same method. He quotes Braun as saying that he used as much as 250 ccm. of the 1-2 per cent. solution at one sitting without sequelae.

Edward Streissler recommends a posterior route with small anterior counter incision for the removal of cervical ribs or dis-ased first ribs.

January 18, 1912.—From the Public Congress of Surgeons, Berlin, Muhsam reports a successful implantation of saphenous vein to replace 6 cm. of urethra removed for impenetrable stric-ture..

No. 12, March 23.—Surgical Society of Breslau. Kuttner re-ports a hernia of the posterior wall of the bladder through the urethra in a 9 week old infant. Recovery following suprapubic opening with hitching up of the posterior bladder wall to the ab-dominal wound.

No. 6, February 10.—Making a ligament out of free periosteal flaps, Katzenstein. He replaced a torn tibio-navicular ligament with a double periosteal implant with reformation of the arch.

No. 11, March 16.—Momburg at Bier's Klinic performed a similar operation through two small incisions, using strips of fascia lata instead of periosteum.

No. 16, April 20.—Incision for Gall Bladder operations. Fritz Konig, of Marburg. The author's incision begins slightly to right of the midline somewhat above the predetermined margin to the liver, passes down curving to the right, ultimately dividing the rectus sheath and muscle directly across, avoiding large ves-sels and nerves. This renders accessible: 1. Gall Bladder; 2. Stomach; 3. Appendix.

The author prints 4 photographs of cases showing post-opera-tive scars. He makes a convincing argument in favor of the in-cision.

A new radical operation for prolapse of the uterus by Hans Hans. This operation is a ventro fixation with a vengeance.

1. Transverse incision.

2. Removal of tubes, upper fundus and mucosa of uterus leaving two lateral "wings."

3. The "wings" are brought out through the peritoneum and between the recti and sewed out flat like two petals of a flower on the external surfaces of the recti.

4. Fascial closure leaving a central opening for through-and-through drainage into the vaginia from the abdominal surface.

No. 18, May 4.—On the technique of paravertebral peripheral anaesthesia. Hans Finsterer succeeded in 4 cases out of six in obtaining anaesthesia sufficient for laparotomy by injecting the nerve trunks close to the vertebral column.

No. 26, June 29.—Supraclavicular anaesthesia. Edward Borchers reports 35 cases of operation on the uppel limbs after novocain-adrenatlin injections into the brachial plexus. Paralysis resulted in all cases of about 4 weeks duration.

No. 29, July 26.—Felix Franke, Treatment of Ecchinococcus Cysts with Formalin. Franke refers to former communications on this subject recommending the following technique: Regardless of the size of cyst: 1. He opens cyte and delivers contents and as much of sac wall as possible. 2. Washes out with 5 per cent. formalin solution in glycerine, up to 100 cc., with or without water. 3. Wipes out free solution and closes cavity without drainage.

Przewalsky calls attention to the maximal gaseous distension of the rectum as a most valuable early sign in appendicitis with peritoitis.

No. 31, July 23.—Carl Springer, in reference to above, states that it was long known in Austria as he got the hint in Hochenegg's Clinic 10 years before.

It is an invaluable sign in children and has been of greatest service to him. He never delays operation when the sign is present.

No. 35, 1912.—Hyperaemia or lymphatic stasis. Gelinsky of Berlin is of the opinion the obstruction to the lymph current is as important as if not more so than that to the blood current in the so-called Hyperaemia of Bier. He gives good general directions on application of the treatment.

